# Reducing neuroendocrine psychosocial stress response through socio-emotional dyadic but not mindfulness online training

**DOI:** 10.3389/fendo.2024.1277929

**Published:** 2024-06-24

**Authors:** Hannah Matthaeus, Christine Heim, Manuel C. Voelkle, Tania Singer

**Affiliations:** ^1^ Social Neuroscience Lab, Max Planck Society, Berlin, Germany; ^2^ Department of Psychology, Humboldt-Universität zu Berlin, Berlin, Germany; ^3^ Institute of Medical Psychology, Charité – Universitätsmedizin Berlin, Berlin, Germany; ^4^ NeuroCure Cluster of Excellence, Charité – Universitätsmedizin Berlin, Berlin, Germany

**Keywords:** hypothalamic-pituitary-adrenal axis, stress, mental training, app-based intervention, randomized controlled trial

## Abstract

**Introduction:**

Stress-related diseases pose significant health risks and show wide prevalence. Empirical evidence suggests that contemplative practices, such as socio-emotional dyadic mental exercises, hold promise in mitigating the adverse effects of stress and promoting psychosocial well-being. This study aimed to investigate the differential effects of two online contemplative mental training programs on the psychosocial stress response: the first involved classic mindfulness practices, while the second incorporated a socio-emotional dyadic approach known as Affect Dyad.

**Methods:**

The study was conducted as part of the longitudinal CovSocial project’s phase 2 in the context of the COVID-19 pandemic. 140 individuals participated in the Trier Social Stress Task (TSST), where the psychosocial stress response was assessed with cortisol saliva samples and subjective stress questionnaires in a cross-sectional design after the active training groups finished their intervention period. Participants were randomly assigned to the socio-emotional training group, mindfulness-based training group, or a control group that did not receive any training. Both training programs consisted of a ten-week intervention period with a daily 12-minute app-based mental training practice and weekly 2-hour online coaching sessions led by mental training teachers.

**Results:**

Results showed that the socio-emotional Dyad group but not the mindfulness-based group exhibited significantly lower cortisol levels at 10, 20, 30, and 40 minutes after the stressor as well as lower total cortisol output compared to the control group during the TSST, indicating a reduced hormonal stress response to a social stressor. Subjective markers did not show differences between the three groups.

**Discussion:**

These findings indicate that the daily socio-emotional dyadic practice, which emphasizes non-judgmental and empathic listening as well as the acceptance of challenging emotions in the presence of others within one's daily life context, may serve as a protective factor against the adverse effects of psychosocial stress triggered by the fear of negative social judgments. Given the high prevalence of stress-related diseases, such online mental training programs based on dyadic practices may thus represent an efficient and scalable approach for stress reduction.

## 1 Introduction

Research has revealed that stress plays a pivotal role in the development of mental disorders (1), and there has been a significant surge in stress-related diseases over the past few decades (2) resulting in substantial costs not only to individual health but also to society as a whole (3, 4). During the COVID-19 pandemic, the resulting psychosocial stressors associated with repeated lockdowns and social isolation led to an acceleration of psychological distress, anxiety, and depression (5). Longitudinal studies found that specifically females, younger people, lower income groups, and people with lower social belonging showed more vulnerable trajectories during prolonged collective stressors which were also associated with higher mental health problems (6, 7). Thus, identifying scalable ways to reduce stress has become of utmost importance for preventing mental health diseases. The CovSocial project has been implemented in the context of the COVID-19 pandemic as a two-phase study. In phase 1, multiple longitudinal markers of vulnerability, resilience, and social cohesion were assessed throughout the different lockdowns and phases of the pandemic in 2020/21 in Germany. As the pattern of results suggested that many participants experienced the pandemic as a major collective stressor negatively affecting their mental health (6, 7), a second phase of the CovSocial project was initiated to investigate if online mental training programs could increase resilience and mental health while decreasing stress and loneliness (8). More specifically, we investigate here whether two types of 10-week online mental training programs, a mindfulness-based and a partner-based socio-emotional dyadic intervention, could reduce psychosocial stress on the neuroendocrine and subjective level.

In humans, the activation of the hypothalamic-pituitary-adrenal (HPA) axis and the subsequent release of cortisol, a steroid hormone, has been consistently demonstrated in response to psychosocial stress situations (9). Prolonged exposure to psychosocial stressors can result in chronic activation of the HPA axis, building up the allostatic load (10). Increased allostatic load has been linked to several negative physical and psychological health outcomes (11). Elevated cortisol reactivity to acute psychosocial stress situations in laboratory settings is associated with heightened stress responses in individuals’ daily lives (12).

One particular approach to address increased stress and related diseases are mindfulness-based interventions, so-called MBIs. Inspired by contemplative traditions from the East and adapted for secular Western healthcare programs, promising results in reducing stress were found in the 8-week program of Mindfulness-Based Stress Reduction (13) and Mindfulness-Based Cognitive Therapy (14). Various other contemplative trainings have experienced a rise in the attention of research on stress reduction programs, thanks to their efficacy in alleviating stress on subjective and neuroendocrine levels (15–17). However, although mindfulness training has consistently demonstrated the ability to reduce stress subjectively (18, 19), the impact of these trainings on physiological stress measures and their buffering effects remains somewhat ambiguous (20–22). Morton et al. (17) argue in their review that differences in findings might be explained by the amount of time that participants practiced outside of a weekly meeting in an 8-week program, as studies have found even small amounts of individual practice to show beneficial results (23). The Monitor and Acceptance Theory (MAT; 24) suggests that the mechanism behind stress reduction through mindfulness training is divided into two steps following each other: First, participants learn attentional and interoceptive capacities (monitoring). Second, they develop emotional capacities, focusing on strengthening a sense of acceptance, which manages the increased receptivity to stress signals (acceptance; 24).

In a 9-month longitudinal mental training study known as the ReSource project (25), three 3-month training modules containing different types of mental practices were compared. The results demonstrated that only the two socially-oriented training modules (socio-affective and socio-cognitive, which incorporated partner-based practices called contemplative Dyads; 26) were associated with reduced cortisol levels in response to stress after the Trier Social Stress Test (TSST; 27). In contrast, the mindfulness-based module, which focused on solitary meditation practices, did not show the same cortisol-reducing effect during the TSST. These results suggest that the partner-based intersubjective practices, involving a daily routine of self-disclosure and the possibility of receiving judgment from the partner, may have contributed to reducing the fear of social judgment. Accordingly, the daily practice of fostering social interactions within a psychologically secure setting of partner-based contemplative dialogues, emphasizing non-judgmental and empathic listening, may potentially serve as a protective mechanism against the deleterious impacts of social stress. As partners would change every week, participants gained a sense of common humanity by sharing their emotions each day with different people and recognizing that everyone’s daily life consists of moments of gratitude and moments of difficult emotions. This effect could extend to experimentally induced psychosocial stressors as encountered in the TSST, which incorporates elements of unpredictability and social threat. It has been suggested that the practice of the Affect Dyad is beneficial for the activation of care- and affiliation-related systems (27, 28), which are linked to positive emotions and capable of reducing threat by inhibiting the activity of the amygdala (29–33). These systems are influenced by oxytocin and opiates (29, 34), which in turn are also involved in stress regulation (35, 36). These mechanisms could explain the successful stress reduction after a 10-week socio-emotional dyadic training. Given that the intensive practice protocol employed in the ReSource project involved several practices within each training module, it was not feasible to isolate and discern the specific effects of a practice type on the stress response. Therefore, in this study, we aim to address this gap by conducting a novel comparison between the direct effects of a daily 12-minute mindfulness practice conducted individually and a 12-minute dyadic practice performed with a partner.

Most MBIs have been developed and are delivered as in-person programs. However, the COVID-19 pandemic has accelerated research on the efficiency of app-based online MBIs, showing a significant beneficial effect in reducing stress, anxiety, and depression during the COVID-19 pandemic (37, 38). The quantity and quality of social interactions predict health and well-being (39), but research focusing on stable, long-term relationships also showed that these can fluctuate throughout the life span (40). A strong trend towards an increase of social interactions via the internet shifted the percentage of social interactions happening online, not only through the spread of smartphones but also as a consequence of the COVID-19 pandemic (41, 42). The beneficial effect of online social interactions depends on their levels of self-disclosure, trust, and perceived support (43, 44). As those qualities can be achieved by partner-based mental training practices, contemplative dyadic practices could function as one approach to these beneficial online interactions.

To test for the specific effects of each type of practice and their efficiency when delivered as a purely online daily mental training program, we compared three groups: One group performed 12 minutes of classic mindfulness practices in the form of an auditory guided meditation with a focus on breathing, listening to sounds or open awareness. The second group practiced a 12-minute socio-emotional dyadic practice, the Affect Dyad (26) in the form of an app-guided contemplative dialogue. The third group served as a control group and received no training. Both active groups conducted an online 10-week training with daily 12-minute practice and weekly 2-hour coaching sessions with mental training teachers (see **Supplementary Material S1**).

Based on previous findings (27), we hypothesized in a preregistration (osf.io/mpr4f) a stronger psychosocial stress reduction in cortisol in the socio-emotional dyadic group as compared to the control and mindfulness group and a stronger psychosocial stress reduction in subjective stress measures in the socio-emotional dyadic group and mindfulness group as compared to the control group.

## 2 Materials and methods

Data were collected as part of phase 2 of the CovSocial project to investigate the differential effects of online mental training programs in a randomized controlled trial (Trial Registration: ClinicalTrials.gov NCT04889508 on May 17^th^, 2020). The CovSocial project is a longitudinal two-phase study to examine mental health during the COVID-19 pandemic in Berlin, Germany (phase 1) and to investigate the effects of two online mental training programs in phase 2. This study was approved by the Charité – Universitätsmedizin Berlin (#EA/199/21) and has been conducted in accordance with the Declaration of Helsinki. The TSST study was preregistered on the Open Science Framework before the first TSST session (osf.io/mpr4f). The present study focuses on hypotheses 1 and 2, but not on the examination of preregistered hypotheses 1a, 2a, 3, and 4, which pertain to individual differences in traits and state trajectories amidst the COVID-19 pandemic, as well as associated mechanisms. These hypotheses will be addressed in a different analysis.

### 2.1 Sample

For the first phase of the CovSocial project, participants were recruited through the Berlin registration office, social media advertisements, and posters within the population of Berlin. The primary criteria for inclusion encompassed an age range of 18 to 65 years, proficiency in the German language for questionnaire completion, and official residency in Berlin during the assessment period. Exclusions were made for individuals not meeting these criteria and for data quality issues, resulting in a final sample of 3,522 individuals for the project’s first three timepoints. In the second phase, participants who had completed assessments during the first three timepoints (T1-T3) of the retrospective longitudinal study in phase 1 (n = 3,522) were invited to undergo prescreening. This screening aimed to determine eligibility for the current intervention study based on specific inclusion and exclusion criteria. Exclusion criteria comprised lack of experience in meditation and yoga practices, absence of educational background in psychology, no presence of psychopathology, suicidality, chronic illness, or pain, and refraining from the use of substances (illegal or prescribed) that could impact physiological stress markers. Furthermore, participants were excluded if their scores on certain questionnaires exceeded designated thresholds: Toronto Alexithymia Scale-20 (TAS-20; 45) scores greater than 60, Patient Health Questionnaire-9 (PHQ-9; 46) scores greater than 19, and Generalized Anxiety Disorder-7 (GAD-7; 47) scores greater than 15. Trained teachers conducted screening calls using the Standardized Assessment of Severity of Personality Disorder (SASPD; 48) and the Composite International Diagnostic Screener (CIDS; 49) to identify and exclude individuals with clinically significant psychopathology. Ultimately, a total of 285 participants were enrolled in the randomized controlled trial (RCT), aligning with the predetermined sample size as previously outlined by Silveira et al. (50).

All participants of phases 1 and 2 underwent a thorough prescreening to assess their suitability for participation in the cross-sectional TSST, conducted at posttest 1 and 2 of phase 2. Recruitment for the TSST included evaluating their medication usage, suicidality, current pregnancy status, hormonal medication usage, and the presence of any endocrine disorders. Individuals who met any of these criteria were excluded from the study based on their responses provided in an online questionnaire. N = 295 participants took part in the prescreening for TSST, of which n = 87 were excluded due to the prescreening criteria (n = 1 study psychology; n = 37 had a meditation routine; n = 1 due to pregnancy; n = 17 had a history of mental disorder; n = 9 suffered from chronic pain; n = 22 due to a score higher than 20 in the PHQ-9 or a score higher than 15 in the GAD-7 or a score higher than 2 at one of the items of the SASPD). Furthermore, n = 68 participants dropped out because it was not possible to make an appointment or they did not show up to their assigned appointment (**Supplementary Figure S1** in **Supplementary Material**).

**Figure 1:**
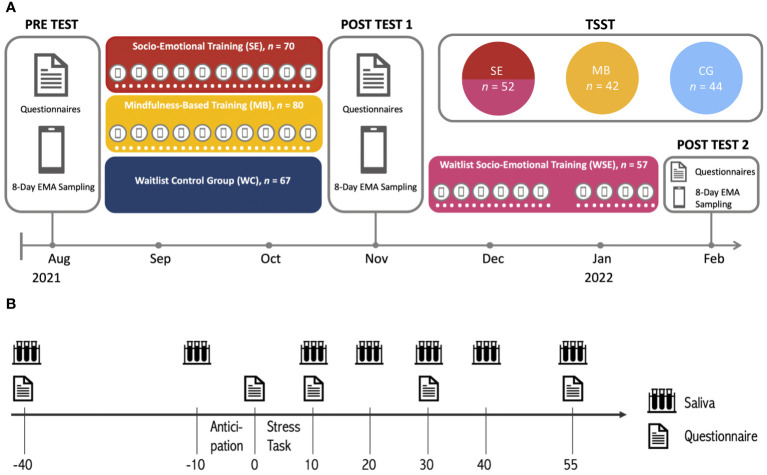
**(A)** Study design of the CovSocial project phase 2 and **(B)** study procedure of the CovSocial phase 2 Trier Social Stress Test (in minutes). The light blue group served as a control group of the TSST only and was not part of the RCT of phase 2 of the CovSocial study.

The TSST was conducted with n = 140 participants (age: M = 44.36, SD = 11.48, range = 18–65, 45 male, 95 female). 94 participants of the intervention conditions (n = 52 in socio-emotional dyadic training, SE; n = 42 in mindfulness-based training, MB) were recruited from phase 2. For the control condition (CG) in the TSST, n = 44 participants of phase 1 were recruited (**Figure 1A**). Two participants dropped out after test instructions were given at -10 min before the stressor. On the testing day, women reported hormonal status via self-report. The groups did not differ in terms of the distribution of participants with varying hormonal status, age, sex, phase of the cycle, alcohol use, smoking, cortisol baseline, anxiety, and depressed mood. 37% of participants were married or cohabiting and had on average 18.2 years of education. Socio-economic status was assessed by household income and employment status. 81% of participants were employed part-time or full-time and 63% had a household income above the average monthly net income in Berlin which is approximately €2175 (51).

Descriptive statistics of the groups can be found in **Table 1**. All participants gave their written informed consent, could withdraw from the study at any time, and were reimbursed at the rate of 15 € per hour.

**Table 1:** Characteristics of TSST participants (n = 138).

	Socio-emotional training (n = 52)	Mindfulness-based training (n = 42)	Control group (n = 44)	*p-*value
Age	45.96 ± 12.02	44.40 ± 11.8	44.09 ± 15.14	.797
Sex (male/female)	12/40	15/27	18/26	.198
Hormonal Status (male/menopause/hormonal contraceptives/natural menstrual cycle	12/17/3/20	15/9/5/13	18/9/2/15	.445
Phase of cycle in women with natural menstrual cycle (follicular/luteal/menstruation/ovulation)	5/7/5/0	5/6/0/1	4/4/3/1	.505
Alcohol use	2.96 ± 2.15	2.92 ± 2.02	3.19 ± 2.07	.962
Smoking	1.85 ± 2.44	2.61 ± 3.59	2.00 ± 2.53	.457
Cortisol baseline (nmol/l)	3.33 ± 2.67	3.31 ± 1.61	4.2 ± 2.02	.085
GAD-7	4.04 ± 3.20	3.06 ± 2.55	3.12 ± 2.56	.164
PHQ-9	4.98 ± 3.29	3.75 ± 3.1	4.35 ± 3.09	.206

*p*-values refer to mean differences across the three groups (ANOVAs) for continuous outcome variables (mean ± standard deviation), and Chi-square tests for categorical data. GAD-7, Generalized Anxiety Disorder 7; PHQ-9, Patient Health Questionnaire-9. All questionnaires were assessed at the pretest of CovSocial phase 2.

### 2.2 CovSocial training program

Both programs consisted of a 10-week mental training with a daily 12-minute practice that was performed via the app and a weekly 2-hour online coaching session that provided further information on the respective training from mental training teachers. In the mindfulness-based group, one of the central techniques utilized was breathing meditation, where participants focused their attention on the sensations of breathing. If their thoughts stray, they were instructed to redirect their attention back to their breath. Additionally, participants engaged in other practices, including attention-based mindfulness of sounds (focusing on sounds in their surroundings) and open-presence meditation (focusing on internal and external sensations). The daily meditation sessions were guided by prerecorded audio delivered via the CovSocial app. The exercise began with participants being prompted to take a comfortable position that induces relaxation while keeping them alert and aware. They were encouraged to be mindful of their current body placement and posture, fostering an attitude of dignity and receptivity towards themselves and their bodies. The primary aim of these practices was to develop present-moment attention and interoceptive body awareness.

The socio-emotional dyadic group was instructed in the Affect Dyad (26), a contemplative mental practice performed with another participant of the group (partners changed every week). The CovSocial app structured the practice in which one partner starts with an exploration of a difficult emotion of the last 24 hours, focusing on how this emotion felt in the body. This was followed by an exploration by the same participant of a moment of gratitude in the last 24 hours and how that experience felt in the body. The listener was instructed to first listen empathically without judgment or any interruption, verbally or non-verbally. In the middle of the practice, roles changed and the listener became the explorer. In the beginning, in the middle, and at the end of the practice partners went into minutes of silence.

The participants engaged in daily practice for six days per week through the CovSocial app. This practice was complemented by weekly 2-hour web-based coaching sessions led by one of four meditation teachers. The coaching aimed to deepen the effects of the practice and integrate it into everyday life. The socio-emotional dyadic training covered dyad ritual, body language, empathic listening, gratitude, dealing with difficult emotions, recognizing patterns in life, and the transfer of the dyad experience to daily life in the coaching sessions. On the other hand, the mindfulness-based training addressed the fundamentals of breathing meditation, body awareness, sensory perceptions, engaging all 5 senses, open awareness, dealing with stress, and the transfer of meditation practice to daily. During the weekly coaching, a combination of short presentations, guided group discussions, and breakout room conversations focused on individual experiences. The content of these presentations was tailored to each specific intervention. For a more detailed description of the intervention protocols, including the onboarding procedures and topics of 10 weekly training sessions for both intervention programs, please refer to **Supplementary Material S1**.

### 2.3 Trier social stress test

The TSST (52) took place shortly after the intervention period for all participants in active training conditions. Due to the potential influence of circadian rhythm on cortisol, testing was conducted between noon and 6 p.m. for 120 minutes. Participants were further asked to refrain from drinking alcohol 24 hours before testing. Two hours before testing they should refrain from doing sports, drinking coffee, eating, smoking, and brushing their teeth. The psychophysiological baseline was assessed upon arrival. Subsequently, 30 minutes of rest was implemented to mitigate any potential immediate stress effects unrelated to the TSST. Following this, participants were given the TSST instructions and asked to assess their subjective stress levels after a 10-minute anticipation period. The stress phase included a 5-minute job interview and a 5-minute arithmetic task. Their performance was mock video-recorded in front of a mixed-sex committee of two alleged behavioral analysts. The jury was trained to remain neutral. After the 10-minute stress phase, participants provided further saliva samples at 10, 20, 30, 40, and 55 min after the stressor started. Subjective stress was assessed at 10, 30, and 55 min (see **Figure 1B**). The testing was closed with a debriefing about the purpose of the test.

### 2.4 Tasks and measures

Seven saliva samples were collected using Salivettes (Sarstedt, Nümbrecht, Germany; 53) to assess free cortisol concentrations (nmol/l) throughout the TSST following the standard protocol. Samples were stored in −30 °C freezers in the laboratory. After study completion, samples were shipped to Dresden LabService GmbH (Germany) for biochemical analysis. At Dresden LabService GmbH, salivates were analyzed using an immunoassay (54). Participants rated their subjective stress experience on the 20-item state scale of the State-Trait Anxiety Inventory (STAI; 55) and the Affect Grid (56), which is a 9x9 grid to assess the current mood on the dimensions of valence and arousal.

### 2.5 Data processing

Cortisol data for 1.2% of the samples were incomplete due to inadequate saliva quantities provided. The missing values in the dataset were handled via Full Information Maximum Likelihood (FIML) estimation implemented in the lme4 package version 1.1.33 (57) in R. Outliers in the cortisol data were identified as values that exceeded the group mean (dyad, mindfulness, control) by more than 3 standard deviations (SD). To reduce the impact of outliers on our results, we replaced them with values equivalent to 3SD above the respective group mean for cortisol (applied to 2.4% of cortisol data) through winsorization across groups. Using the winsorized cortisol data, we calculated the area under the curve with respect to ground (AUCg) across the complete time course of the study to determine total cortisol output. Cortisol data were transformed using the Yeo-Johnson transformation (58) with a λ value of -0.44, using the caret package version 6.0 (59).

### 2.6 Statistical analysis

Variables with potential influence on hormonal stress responses were assessed in all participants and used as covariates in all analyses that tested for group differences. Covariates included age, sex, hormonal status, phase of the menstrual cycle, number of smokers, anxiety, and depressed mood, using Pearson’s Chi-squared tests and one-way analyses of variance (ANOVAs) (see **Table 1**). The main analyses were performed in R (version 4.0.2; 60) using the lme4 package version 1.1.33 (57). All analyses were Bonferroni corrected and used alpha = .05 as the significance threshold. Changes in cortisol over the 95 minutes of the TSST were analyzed via a multilevel growth curve approach including the independent variable group (socio-emotional, mindfulness-based, control) and the interaction of group and time. We included linear, quadratic, and cubic effects of time in our modeling. The building of these models followed a stepwise approach, and we assessed their overall fit by comparing nested models using the log-likelihood ratio. Individual baseline differences were considered by random intercepts, while random slopes capture differences in individual trajectories over time (61). Hormonal status, time of day, and age were added as control variables to the cortisol model due to their potential influence on the HPA axis response. Bonferroni corrected *post-hoc* comparisons were used to test for group differences at all seven timepoints. Further, we compared the three groups in their total cortisol output (AUGg) using Bonferroni corrected *post-hoc* ANOVAs. The same analyses were applied for subjective stress measures with age and sex as covariates.

## 3 Results

Based on the baseline-to-peak increase in percentage as a measure of cortisol responder rate (62), all participants were classified as responders, while using the 1.5nmol/l criterion (62) identified 70% of participants as responders.

The baseline linear growth curve model was improved by incorporating random intercepts, random slopes, a quadratic and cubic trend of the time, a main effect of group, group-by-time interactions, and covariates such as hormonal status, age, and time of day, resulting in enhanced model fit. The model with a polynomial term for time showed a significantly better fit than the null model with only an intercept (deviance = 740.95, df = 2, p < 0.001). The REML log-likelihood of the improved model was -988.2. The coefficients of the final model can be retrieved from **Table 2**.

**Table 2:** Coefficients of the growth curve model (total number of observations: 966; number of participants: 138) predicting cortisol changes over time by group (socio-emotional group, mindfulness-based group, control group).

Fixed Effects	b	SE	
Baseline level β_0_	1.28 ***	0.08	
Time linear, β_1_	2.02 ***	0.46	
Time quadratic, β_2_	-0.68 **	0.21	
Time cubic, β_3_	-2.33 ***	0.25	
Group_mindfulness_, β_0,gmind_	-0.07	0.04	
Time linear by Group_mindfulness_, β_1,gmind_	0.16	0.66	
Time quadratic by Group_mindfulness_, β_2,gmind_	0.23	0.30	
Time cubic by Group_mindfulness_, β_3,gmind_	-0.09	0.36	
Group_dyad_, β_0,gdyad_	-0.12 **	0.04	
Time linear by Group_dyad_, β_1,gdyad_	-0.31	0.62	
Time quadratic by Group_dyad_, β_2,gdyad_	0.21	0.29	
Time cubic by Group_dyad_, β_3,gdyad_	0.27	0.35	
Random effects	b	SD	Covariance baseline-slope
Variance baseline level, b_0i_	0.04	0.19	–
Variance slope linear, b_1.1i_	8.08	2.84	0.45
Variance slope quadratic, b_1.2i_	0.77	0.88	-0.36
Variance slope cubic, b_1.3i_	1.64	1.28	-0.40
Residual, ϵ_ti_	0.01	0.09	–

The displayed model uses Yeo Johnson transformed values. SE, standard error; SD, standard deviation. - not applicable, **p <.01, ***p <.001.

**Figure 2:**
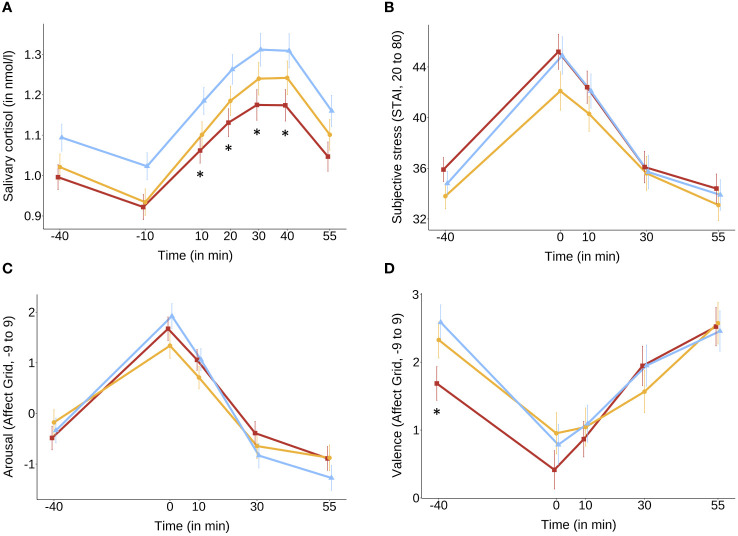
Mean scores and SE bars extracted from linear mixed models with hormonal status, age and time of day as covariates of **(A)** salivary cortisol and with sex and age as covariates of **(B)** subjective stress levels (STAI), **(C)** subjective arousal (Affect Grid) and **(D)** subjective valence (Affect Grid) for socio-emotional (red), mindfulness (yellow) and control group (blue) over time of the TSST. *p <.05 (socio-emotional *vs*. control group for each timepoint). Salivary cortisol was reduced compared to the control group at timepoints +10, +20, +30, and +40 minutes after stressor onset **(A)**. No differences between training conditions after stressor onset were found in subjective stress **(B–D)**.

Significant differences in mean cortisol concentrations (**Figure 2**) were observed for SE compared to CG at timepoint +10 (*Δmean* = 0.12, *p* = .012), +20 (*Δmean* = 0.13, *p* = .019), +30 (*Δmean* = 0.14, *p* = .033) and +40 (*Δmean* = 0.13, *p* = .047).^1^ We found significantly higher total cortisol output (AUCg) in CG as compared with the SE (*Δmean* = 11.29, *p* = .009), but not between CG and MB (*Δmean* = 7.32, *p* = .182) as well as between MB and SE (*Δmean* = 3.97, *p* = .875). Compared to the control group, the percentage reduction in the baseline-to-peak increase (Δcortisol) was 36% for dyad training (*t*_dyad_ = 2.28, *p* = .025). The change in mean cortisol concentrations from baseline to peak exhibited a substantial effect size of *d* = 1.00.

The inclusion of a cubic time trend led to significant increases in the model fit of the subjective stress models. There were no significant group differences found for subjective stress ratings (STAI), arousal, and valence (both measured with the Affect Grid) in the growth curve model or total cortisol output (AUCg). Results of the model comparisons and coefficients of the final models are displayed in the **Supplementary Material Tables S1**-**S3**.

*Post-hoc* analyses showed no significant differences between the groups in early life adversity assessed with the Childhood Trauma Questionnaire (CTQ; 63; *Δmean_MB_CG_* = -0.53, *p* = 1; *Δmean_SE_CG_* = -0.94, *p* = 1; *Δmean_SE_MB_* = -0.42, *p* = 1), chronic stress levels assessed with the Trier Inventory for the Assessment of Chronic Stress (TICS; 64; *Δmean_MB_CG_* = 0.26, *p* = 1; *Δmean_SE_CG_* = -3.36, *p* = .145; *Δmean_SE_MB_* = -3.93, *p* = .111), and general trait-vulnerability assessed in phase 1 (6; *Δmean_MB_CG_* = 0.01, *p* = 1; *Δmean_SE_CG_* = -0.25, *p* = .280; *Δmean_SE_MB_* = -0.26, *p* = .244).

## 4 Discussion

The main goal of this intervention study was to examine the differential effects of two online mental training programs on their ability to reduce the stress response to a social stressor: A classic mindfulness-based program (MB) with solitary meditation practices was compared to a socio-emotional dyadic program (SE) based on the Affect Dyad (26), which is performed together with a partner. Participants practiced 12 minutes daily via an app and were coached by teachers in weekly 2-hour online sessions. Upon completion of the 10-week intervention program, participants underwent a TSST (52) to assess their subjective and neuroendocrine stress responses. In the TSST, both groups were compared to a control group (CG) without any training. The main aim was to determine whether the short, low-dose, and thus scalable daily online interventions could reduce the psychosocial stress response.

As expected, SE showed significantly lower cortisol levels compared to CG at 10, 20, 30, and 40 minutes after the stressor of the TSST, indicating a reduced stress response at the peak cortisol timepoints. Additionally, SE exhibited a significantly lower total cortisol output (AUCg) as compared to CG. Comparisons between MB and CG did not show significant differences in cortisol levels or total cortisol output as well as the direct comparison between the MB and SE.

This study significantly expands upon previous research by addressing a key limitation observed in the ReSource project. In the original project, the 9-month, in-person ReSource protocol did not permit the isolation of the specific effects of the Affect Dyad practice alone. This was due to the comprehensive nature of the protocol, which included four days of introductory retreats for each of the three 3-month modules, along with weekly 2-hour, in-person practice sessions led by mental training teachers, and the completion of two daily practice sessions via a designated app. Consequently, participants were exposed to a variety of practices within each training module. In this study, we took a novel approach by directly comparing the daily practice of the Affect Dyad with a classic mindfulness practice of similar duration. This allowed us to discern, for the first time, the unique impact of the Affect Dyad practice. Our findings indicate that the socio-emotional dyadic exercise was particularly effective in reducing psychosocial stress responses at the hormonal cortisol level. As compared to classic mindfulness practices, the socio-emotional dyadic practice reduced the cortisol response to a social lab stressor by 36%. This finding aligns with prior results from the ReSource project, where the Affect Dyad, among other contemplative practices, was part of a three-month daily training module. After six months of consistent practice with this module, alongside another three-month module, participants of the socio-emotional training exhibited a 48% reduction in cortisol response to stress during the TSST compared to the control group (27). Results from the ReSource project revealed that after three months of intense training, the socio-emotional training significantly differed from both the mindfulness group and the control group. Following a 6-month mindfulness-based and socio-cognitive training, participants experienced a 51% reduction in cortisol levels from baseline to peak (27). In comparison, this study observed a 36% reduction, potentially attributable to the shorter duration of training and less comprehensive training protocol. Since the primary aim was to assess the generalizability of ReSource project findings to a less intense training protocol, the results are promising, demonstrating a significant distinction between socio-emotional training and the control group.

Given that low cortisol can also indicate a heightened vulnerability associated with a blunted stress response, which is considered detrimental (65–67), we investigated potential distinctions among the three groups within our study’s overall healthy TSST sample. We focused on factors such as early life adversity, chronic stress levels, and the trajectory of vulnerability during the COVID-19 pandemic, which served as the backdrop for our investigation. As no significant differences were found, it is plausible to assume that the lower cortisol response observed in the dyad group indicates a health-related beneficial effect.

The effectiveness of the Affect Dyad practice in stress reduction may stem from its incorporation of various contemplative elements known for their significant benefits in enhancing mental well-being and mitigating stress.

Participating in daily sessions where individuals share intimate feelings and sensations with a stranger, while also engaging in empathic listening as their partner opens up about personal emotions, is believed to evoke a profound sense of common humanity. The practice nurtures the understanding that everyone faces challenges in their daily lives. Consequently, as participants continue to practice sharing their inner feelings with strangers and engage in empathic listening within a psychologically safe environment, the fear of negative judgment from others is likely to diminish over time. This transformation occurs through the establishment of trust between both partners, fostering an atmosphere where judgment is suspended, and open communication thrives. Simultaneously, participants develop the skill of attentively observing their bodily sensations and learning to correlate them with the emotions they experience in response to their surroundings. This process fosters a deeper connection with the body as well as acceptance of challenging emotions. Therefore, such intersubjective dyadic practices and specific socio-emotional ones may be particularly efficient in reducing social stress, one of the most prominent causes of stress-related diseases (68–70).

In contrast to our expectations, we did not find differences between groups in the subjective stress responses assessed with the STAI and the Affect Grid. This outcome diverges from an extensive body of literature demonstrating reductions in subjective stress through mindfulness training (19) and other socio-emotional training programs (27) and raises questions about the factors contributing to the non-replication of these established findings within the present study. One explanation may be the unreliability of the self-report. It is important to acknowledge, however, that across all experimental groups, the STAI exhibited meaningful elevations after the initiation of the stress-inducing stimulus, coupled with plausible recovery phases thereafter. A more plausible explanation may be that the STAI, which is rather an anxiety index than a direct stress measure (55), might not optimally capture the training-induced diminution of stress-associated responses. The usage of more profound stress self-report measures may be more adequate for future stress-related research.

Further, self-report measures have previously been shown to be less sensitive to depict differences between mental practice types as compared to their objective counterpart measures (16, 71). In line with such a view, Engert et al. (27) observed clear differential findings in the cortisol output between different training modules in the TSST, but no differential findings between training modules when looking at self-report measures only. Another factor may have been that our study utilized a relatively short ten-week training period with a daily 12-minute practice. A longer training duration might have yielded more pronounced subjective stress reduction effects. Finally, it is important to acknowledge that subjective stress measures and objective stress measures often exhibit only moderate correlations even at baseline (72–74). Thus, self-rated stress effects may not always align with physiological stress responses in training contexts as well (75). While cortisol levels serve as objective biomarkers of stress (73), subjective experiences of stress can be influenced by a range of factors beyond physiological reactivity (76). Psychological factors, individual differences in stress perception, and the complex interplay between mind and body could contribute to the discrepancies between subjective and physiological stress responses observed in this study (74, 77). Future studies will have to further explore the relationship between different subjective and objective stress-related measures at baseline (75) and after training.

Overall, our findings confirm that we can indeed reduce the endocrine stress response as measured through cortisol levels in saliva throughout a laboratory psychosocial stressor task after 10 weeks of daily 12-minute partner-based dyadic socio-emotional training accompanied by weekly coaching sessions led by trained mental training teachers. Additionally, the app-based dyadic approach helped compliance with daily practice given the social requirement and the wish not to let the respective partner down. Accordingly, in another paper of our group, we show that compliance to the 10-week dyadic practice was higher than to mindfulness practice done alone (50, 78).

Potential mediators of these effects were not explored in this study. However, Silveira et al. (50) found in the same study that both interventions, SE and MB, led to higher levels of empathy and compassion for self and others. Only after the socio-emotional Dyad training though, the observed changes in self-compassion were moderated by an increase in acceptance. This may point to the importance of future research investigating the association of compassion towards self and others as well as acceptance with stress reduction. Self-compassion consists of self-kindness, common humanity, and mindfulness on the one hand and less self-judgment, isolation, and overidentification on the other hand (79). Those factors are hypothesized to be specifically reduced through the Affect Dyad, as participants learn to accept difficult emotions as well as to focus on gratitude in the presence of another person who is trained not to judge while listening. Furthermore, rotating partners weekly fosters tolerance and a sense of common humanity, as it underscores the universal desire among individuals to alleviate suffering and pursue happiness. Thus, this increase in self- and other-related compassion may explain the beneficial effect of this interpersonal practice in stress reduction. In the context of mindful self-compassion interventions (80), future research could compare whether the specific components of self-compassion are equally contributing to stress reduction after self-compassion and Dyadic interventions.

When it comes to potential underlying mechanisms for observed stress reduction on a biological level, several transmitters may be involved. More generally, based on previous studies, we suggest that socio-emotional compassion-based training may activate biological care and affiliative systems (27, 30), a system that is associated with the release of oxytocin. Indeed, the release of oxytocin has been widely associated with beneficial health outcomes (36). The impact of oxytocin on stress reduction lies primarily in its effects on social behavior as it is released in response to social stimuli which can promote stress reduction (81). This mechanism is modulated by the HPA axis and the inhibition of cortisol release through oxytocin. When assessed in the context of a socio-emotional practice for 3 months within an intense in-person and app-based training program with 30 minutes of daily practice, oxytocin was found to show mixed results. Engert et al. (82) argue that a complex interplay between oxytocin and a cortisol stress response might rather lead to a recovery-boosting than a reactivity-buffering effect by oxytocin release in a psychosocial stress test. Furthermore, socio-emotional training led to decreased levels of overall oxytocin levels in a psychosocial stress situation, but those changes were not related to differential cortisol release or subjective stress measures (83). These findings suggest that stress reduction through oxytocin is modulated by complex interplays and while it may not directly mediate stress reduction, it might be modulating the emotional saliency of stressor cues as well as regulating stress-reducing behavior. Further research is necessary to elicit the detailed mechanisms underlying oxytocin’s effects on stress regulation.

The limitations of this study should be acknowledged to contextualize the interpretation of the results. Firstly, the relatively short duration of the intervention, spanning only 10 weeks, may have limited the magnitude of observable effects, especially in subjective stress measures which may require longer durations to manifest noticeable changes. Additionally, the sample sizes of the intervention groups were relatively small, which could have hindered the detection of significant differences. The study did not explore potential long-term effects beyond the immediate post-intervention period, thus limiting the understanding of sustained benefits or potential adverse outcomes. Finally, it’s important to note that the TSST cannot easily and reliably be administered twice due to the necessity of debriefing afterward for ethical reasons and its reliance on the element of surprise, which is essential to its effectiveness. The test’s efficacy stems from participants’ unawareness of what to expect, which induces a genuine stress response. Therefore, while our comparison with a control group enables conclusions about the training-related effects of the two mindfulness practices, establishing strict causality is challenging without a pre-intervention TSST measure.

In conclusion, our study provides evidence that a daily 12-minute, online socio-emotional dyadic mental training program performed over 10 weeks and supported by weekly teacher-based online coaching can effectively reduce a cortisol response to psychosocial stress. Given the continuously increasing stress-related diseases (2, 84), finding scalable mental training approaches for effective stress reduction became a global and urgent goal. The present finding can inform the development of scalable intervention formats that help promote mental well-being and reduce psychosocial stress in daily life. It underlines the importance of incorporating intersubjective dyadic practices into classical MBIs.

## Data availability statement

The datasets presented in this article are not readily available. The raw data sets generated and analyzed during this study are not publicly available because of proprietary rights and data protection policies but are available from the corresponding author upon reasonable request. Requests to access the datasets should be directed to hannah.matthaeus@social.mpg.de.

## Ethics statement

The studies involving humans were approved by Ethics Committee of Charité – Universitätsmedizin Berlin (#EA/199/21). The studies were conducted in accordance with the local legislation and institutional requirements. The participants provided their written informed consent to participate in this study.

## Author contributions

HM: Conceptualization, Data curation, Formal analysis, Investigation, Methodology, Writing – original draft, Writing – review & editing. CH: Conceptualization, Methodology, Supervision, Writing – review & editing. MV: Conceptualization, Formal analysis, Methodology, Supervision, Writing – review & editing. TS: Conceptualization, Data curation, Funding acquisition, Investigation, Methodology, Project administration, Resources, Supervision, Writing – review & editing.

## Footnotes

1 All comparisons of each timepoint per group can be found in the **Supplementary Material**.
